# *Euphorbia tirucalli* latex loaded polymer nanoparticles: Synthesis, characterization, *in vitro* release and *in vivo* antinociceptive action

**DOI:** 10.1371/journal.pone.0274432

**Published:** 2022-11-29

**Authors:** Marina Lima Rodrigues, Anderson de Jesus Gomes, Mani Indiana Funez, Mariane Aparecida da Silva Marques, Claure Nain Lunardi

**Affiliations:** 1 Laboratory of Photochemistry and Nanobiotechnology, University of Brasilia, Campus Ceilandia, Brasília, Federal District, Brazil; 2 Program in Nanoscience and Nanobiotechnology, University of Brasilia, Brasília, Federal District, Brazil; 3 Sciences and Technologies in Health Program, University of Brasilia, Campus Ceilandia, Brasilia, Federal District, Brazil; Siksha O Anusandhan University School of Pharmaceutical Sciences, INDIA

## Abstract

The encapsulation of drugs in micro and nanocarriers has helped to resolve mechanisms of cellular resistance and decrease drug side effects as well. In this study, poly(D,L-lactide-co-glycolide) (PLGA) was used to encapsulate the Euphol active substance-containing latex from *Euphorbia tirucalli* (E-latex). The nanoparticles (NP) were prepared using the solvent evaporation method and the physical and chemical properties were evaluated using spectrophotometric techniques. FTIR was used to prove the formation of the ester bond between the E-latex and PLGA-NP. The UV-Vis spectroscopic technique was used to show that more than 75% of the latex was encapsulated; the same technique was used to determine the release profile of the compound at different pH values, as well as determining the speed with which the process occurs through kinetic models, and it was observed that the best adjustments occurred for the Korsmeyer-Peppas model and the Higuchi model. The DLS technique was used to determine the diameter of the particles produced as well as their zeta potential (ZP). The sizes of the particles varied from 497 to 764 nm, and it was observed that the increase in E-latex concentration causes a reduction in the diameter of the NP and an increase in the ZP (-1.44 to -22.7 mV), due to more functional groups from latex film being adsorbed to the NPs surfaces. The thermogravimetric experiments exhibit the glass transition temperatures (Tg) that is appropriate for the use of formulated NPs as a stable drug delivery device before use. The *in vivo* activity of E-NPs (30 and 100 mg/Kg/p.o.) was tested against carrageenan-induced mechanical hypernociception. The data demonstrated a significantly antinociceptive effect for E-NPs, suggesting that E-latex nanoencapsulation preserved its desired properties.

## Introduction

Several uses of Euphorbia latex (E-latex) include as an antitumor [1], immunodulator [2], antimicrobial [3], antitumoral [4], as well as molluscic activity [5] and a potential source of biofuel [6]. The plant produces milk sap containing euphol, phorbol diterpene esters, tiglians, daphananas, aromatic and ingenuous daphans. organic substances are also present [7]. In Euphorbia sap and latex, tetracyclene alcohol is present; it has anti-inflammatory, contraceptive, and anti-fertility properties [8, 9]. The latex is investigated in high dilutions for cancer and AIDS showing that it modifies glycolytic viability and non-tumor melanocytes; breast cancer cells showed an efficient reduction in tumor growth in rats [10]. However, the greatest downside to treating cancer is the long-term side effects on both healthy cells and cancerous cells [11]. Another side effect of cancer treatment is pain. Pain is a frequent symptom at diagnosis and during cancer treatment [12, 13]. It could be related to disease or its management. There are distinct cancer-related pains [13]. It is often disabling, causing significant impairments in a patient’s quality of life and its treatment remains challenging.

Nanocarriers used for medical applications must be biocompatible, able to integrate with a biological system without eliciting an immune response or any negative effects and they must be nontoxic (harmless to a given biological system) [14–18]. In this work, PLGA nanoparticles (NPs) were produced containing E-latex; this biodegradable copolymer undergoes hydrolysis in the body, producing biodegradable metabolite monomers, such as lactic acid and glycolic acid, resulting subsequently in CO_2_ and H_2_O via the Krebs cycle [19–23]. Thus, in this study we characterized PLGA NPs loaded with E-latex (E-NP) and evaluated its antinociceptive action in an animal model [24].

## Materials and methods

### Materials

Poly(D,L-lactide-co-glycolide) (lactide:glycolide 50:50, mol wt. 30,000–60,000), poly(vinyl alcohol) (87–90% hydrolyzed, average mol wt. 30,000–70,000), dichloromethane (analytical standard), methyl alcohol anhydrous (99.8%), ethyl alcohol (99.5%), dimethyl sulfoxide anhydrous (99.9%), carrageenan and phosphate buffered saline (PBS) were obtained from Sigma-Aldrich^®^ (St Louis, MO, USA). Distilled water of Milli-Q quality was used. The *Euphorbia Tirucalli* latex was extracted in our laboratory.

### Latex of Euphorbia tirucalli

The latex of *Euphorbia tirucalli* (E-latex) was collected at 15.8314° S and 48.1142° W at 1170 m altitude (Brasília-DF, Brazil). To mitigate component degradation, the latex was immediately transferred to vials and frozen. To improve the quantification and accuracy of the results, the frozen latex was powdered; 12 mL of latex was collected and solubilized in 38 mL of methanol, left to stir for 3 days to obtain the powder and stored in the absence of light [25].

### Calibration curve

The analytical curve was made by dissolving 1.0, 2.0, 3.0, 4.0, and 5.0 mg of latex powder in 10 mL of methanol. All solutions (n = 5) had their absorbance values measured spectrophotometrically at a wavelength of 205 nm (Lambda 25 Perkin Elmer spectrophotometer). The obtained results led to the construction of the line equation obtained through linear regression and the analytical curve.

### E-loaded PLGA nanoparticles (E-NPs)

**Fig 1:**
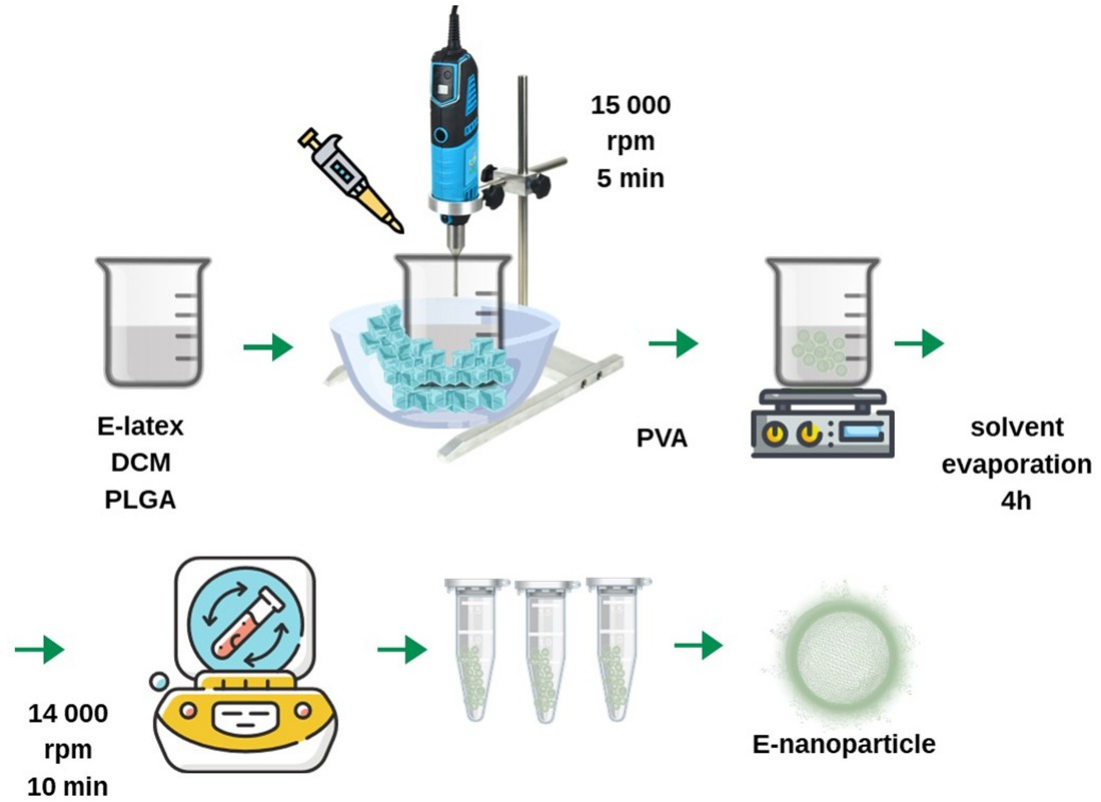
Steps of the preparation of blank NPs and E-NPs using the solvent evaporation method.

A method for solvent evaporation was chosen, and blank NPs and E-NPs were prepared with the *Euphorbia tirucalli* latex powder. Two solutions were used in the preparation of NPs, the first solution being polyvinyl alcohol (PVA) 2.0%. The second solution was prepared by dissolving 60 mL of dichloromethane (CH_2_Cl_2_) in 0.05 g of PLGA [26, 27]. The aqueous solution of 2.0% PVA was carried to a mechanical homogenizer and the organic solution of PLGA was slowly dripped. The solution was then stirred (magnetic stirring) for 4 hours to allow the dichloromethane to evaporate. This substance was centrifuged for 5 min at 15000 rpm, then the PVA-containing the supernatant was extracted to check the percentage of the encapsulated compound in the spectrophotometer [28]. Precipitated particles were rinsed, with 1.0 mL of sterile water three times. The samples were refrigerated at 2.0°C for storage and then freeze-dried. Fig 1 illustrates the production of the E-NPs.

### Encapsulation efficiency (EE%)

The amount of E-latex in the E-NP was calculated using the indirect process, which included separating the supernatants of all preparations, diluting by 100 times, and quantifying using a previously validated calibration curve. The percentage ratio of the amount of drug associated with the nanoparticles to the initial amount of drug used to produce the particles in triplicates was defined as the efficiency of encapsulation using the values obtained from Eq 1.


$$EE\left(\%\right)=\frac{amount\;of\;encapsulated\;drug}{initial\;amount\;of\;drug}\times 100$$ (1)

### Size distribution and zeta potential

A Zetasizer Nano ZS was used to determine the nanoparticles, size distribution and zeta potentials (Malvern Instruments Ltd., Worcestershire, United Kingdom). The dynamic light scattering mode (DLS) was used to measure the sizes of 100 times diluted E-NPs and blank-NPs. The dispersant used in the study was phosphate-buffered saline (PBS 10 mM). For size analysis, each sample was measured three times with ten runs each time. The Laser Doppler electrophoresis mode was used to determine the zeta potential of each sample, with at least 10 runs at a constant temperature (25°C). Three independent tests yielded size average and zeta potential. Unless otherwise mentioned, all DLS sizes, PdI, and zeta potentials were calculated after dilution [29].

### Fourier Transform Infrared analysis

A Fourier Transform Infrared (FTIR) spectrophotometer, model IR Prestige 21 (Shimadzu^®^) was used to obtain spectra for E-latex, blank-NPs, and E-NPs. For the FTIR assay, each sample was first combined with potassium bromide (KBr) at a ratio of 1:10 mg and compressed into a tablet. The detection wavelength was between 400 and 4000 cm^-1^, and there were 40 scans at a resolution of 4.0 cm^-1^ [30].

### Differential Scanning Calorimetry (DSC)

The physical state of the E-latex inside the NPs was characterized by the analysis of the DSC curves. The curves were obtained in a DSC cell (Shimadzu^®^ model DSC-60A) using aluminum crucibles with about 3.0 mg of samples, which were subsequently sealed with a press. The analyses were performed under a dynamic nitrogen atmosphere at 20 mL×min^-1^ and heating rate of 5.0°C×min^-1^, in the temperature range of 35 to 400°C. The DSC equipment was pre-calibrated with metal indium (purity above 99.99%, melting temperature = 156.4°C). The data obtained from the thermal events of the samples were identified in the curves obtained by the software TA-60WS^®^.

### Simultaneous TG-DTA

The thermal stability of the samples was verified by simultaneous TG and DTA, using a Shimadzu DTG-60A^®^, using aluminum crucibles with about 3.0 mg of samples, a dynamic nitrogen atmosphere of 50 mL×min^-1^ and a heating rate of 5.0°C×min^-1^ in the temperature range of 30 to 400°C.

### *In vitro* drug release and kinetic analysis

At 37°C the 10 mg of E-NPs was dispersed in 3.0 mL PBS (pH 1.0, 7.4 and 8.5) and UV-Vis absorption values at 205 nm were measured at predetermined time intervals until the release plateau was reached (t = 6 hours). For the kinetic experiments, KinetDS 3.0 software was used; the equations were chosen from the most popular mechanistic and empirical models applied to the drug release curve such as: zero-, first-, second- and third-order, the kinetic models of Higuchi, Korsmeyer-Peppas, Weibull, Hixson-Crowell and the Hill equation [31, 32].

$$Zero\text{-}order\;model:Q_{t}=Q_{0}+k_{0}\times t$$ (2)

$$First\text{-}order\;model:logQ_{t}=logQ_{0}+\frac{k\times t}{2.303}$$ (3)

$$Pseudo\text{-}first\text{-}order:\text{ln}\;\left(Q_{e}-Q_{t}\right)=lnQ_{e}-k_{1}\times t$$ (4)

$$Second\text{-}order\;model:\frac{1}{Q_{t}}=\frac{1}{Q_{0}}-k_{2}\times t$$ (5)

$$Pseudo\text{-}second\text{-}order\;model\;(type\;I):\frac{t}{Q_{t}}=\frac{1}{k_{2}Q_{e}^{2}}+\frac{t}{Q_{e}}$$ (6)

$$Pseudo\text{-}second\text{-}order\;model\;(type\;II):\frac{t}{Q_{t}}=\left(\frac{1}{k_{2}Q_{e}^{2}}\right)\frac{1}{t}+\frac{t}{Q_{e}}$$ (7)

$$Pseudo\text{-}second\text{-}order\;model\;(type\;III):Q_{t}=Q_{e}-\left(\frac{1}{k_{2}Q_{e}}\right)\frac{Q_{t}}{t}$$ (8)

$$Pseudo\text{-}second\text{-}order\;model\;(type\;IV):\frac{Q_{t}}{t}=k_{2}Q_{e}^{2}-k_{2}Q_{e}Q_{t}$$ (9)

$$Third\text{-}order\;model:\frac{1}{Q_{t}}=\frac{1}{Q_{0}^{2}}-k_{3}\times t$$ (10)

$$Higuchi\;model:Q_{t}=k_{H}\times \sqrt{t}$$ (11)

$$Korsmeyer\text{-}Peppas\;model:Q_{t}=k_{KP}\times t^{n}$$ (12)

$$Weibull\;model:Q_{t}=1-exp\;(\frac{-{\left(t-T_{i}\right)}^{b}}{a})$$ (13)

$$Hixson\text{-}Crowell\;model:\sqrt[{3}]{Q_{0}}-\sqrt[{3}]{Q_{t}}=k_{HC}\times t$$ (14)

where *t* is the time, Q_t_ is the amount of drug released at time *t*, Q_0_ is the initial amount of the drug in the nanoparticles, k_0_ is the zero-order rate constant, Qe is the amount of the drug at equilibrium, k_1_ is the first-order rate constant, k_2_ is the second-order rate constant, k_3_ is third-order rate constant, k_H_ is the Higuchi constant reflecting the design variables of the system, k_HC_ is the rate constant for the Hixson-Crowell rate equation, k_KP_ is the rate constant in Korsmeyer-Peppas model equation and *n* is the release exponent, indicative of the drug release mechanism. The accuracy of these models was compared by calculation of squared correlation coefficient (r^2^). In addition, the Korsmeyer-Peppas model is employed to characterize drug release mechanisms: Fickian release (diffusion-controlled release), non-Fickian release (anomalous transport) and case-II transport (relaxation-controlled release). When *n* ≤0.43, it is Fickian release. An *n* value between 0.43 and 0.85 is defined as non-Fickian release. When *n* ≥ 0.85, it is case-II transport [33]. In the Weibull model, *Ti* is the lag time between the initial of measurement and the release of the drug (in most cases *Ti* = 0), *a* is the time scale of the process and *b* is the shape parameter (the shape of the release curve is exponential if b = 1, parabola if b < 1 or sigmoid if b > 1) [34].

### *In vivo* hypernociception assay

The experiments were performed on male Wistar rats (150–300 g) housed in standard clear plastic cages (four per cage) with free access to food and water. All behavioral testing was performed between 9:00 am and 5:00 pm in a temperature-controlled room. Animal care and handling procedures were in accordance with the International Association for Study of Pain (IASP) Guidelines [35], the Guide for the Care and Use of Laboratory Animals of the National Council for the Control of Animal Experimentation (CONCEA, Brazil) and the Guide for the Care and Use of Laboratory Animals of the Institute for Laboratory Animal Research [36]. All efforts were made to minimize the number of animals used and their suffering. The study was approved by the Animal Research Ethics Committee of the University of Brasília (Protocol 58/2019) and the registry in the National System for Genetic Heritage and Associated Traditional Knowledge Management (SisGen)-A906C8F.

### Nociceptive mechanical test

Mechanical hypernociception was evaluated in rats using Dynamic Plantar Aesthesiometer (Ugo Basile) [37]. This pressure transducer is coupled to a digital force detector that measures the applied force in grams. Prior to starting the experiment, rats were placed in acrylic cages with wire grid floors for 15–30 minutes, which enabled the rats to begin environmental adaptation. The test employs a filament-containing universal tip, which applies upward pressure on the rat’s hind paw, to evoke a hind paw flexion reflex. Once the paw was pulled away, the rat immediately flinched. Via paw removal, the registered force was automatically displayed. The findings are expressed as hypernociception strength (in grams), which is determined by subtracting the force measured after treatment from the basal value (O force).

### Drug preparation, administration, and experimental protocols

**Fig 2:**
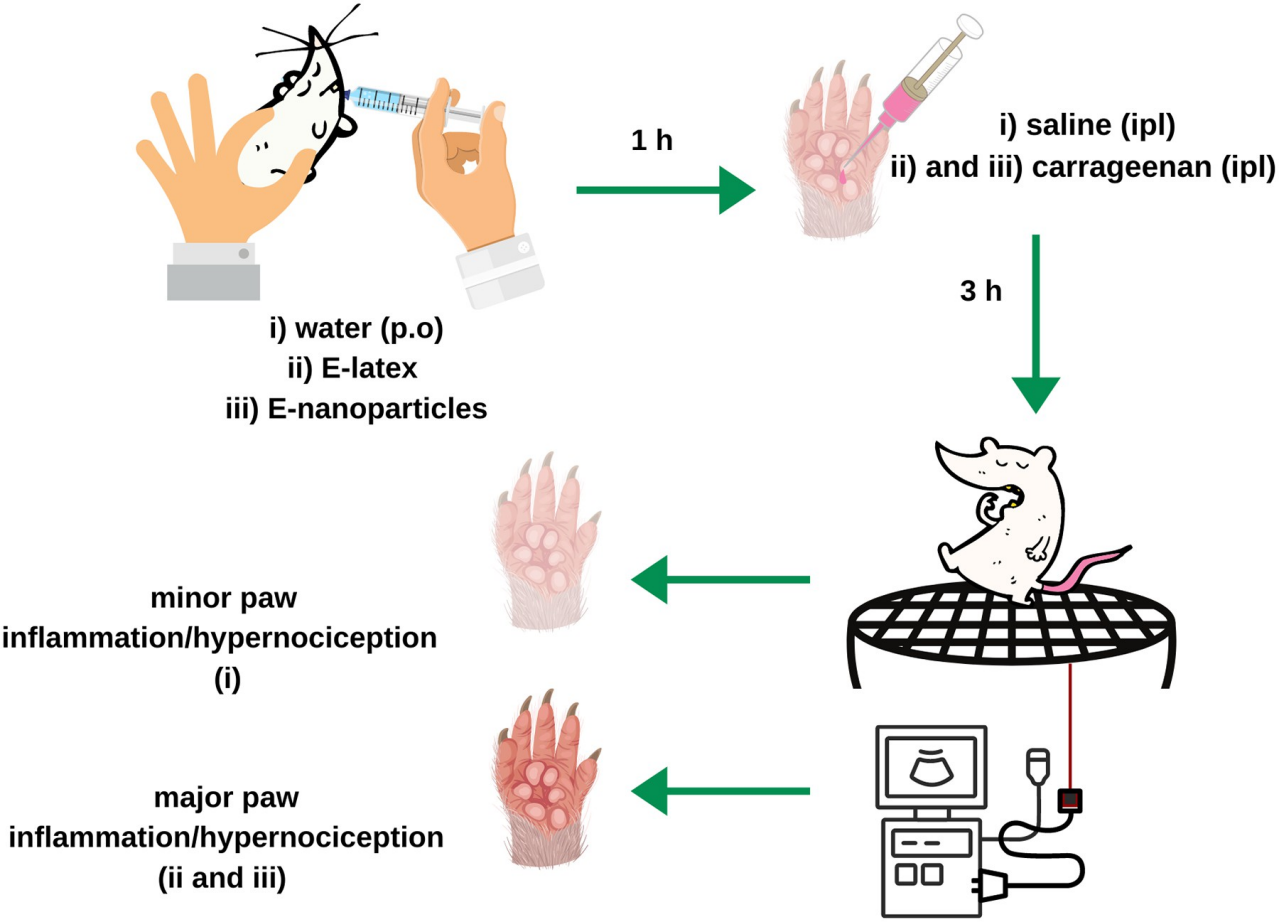
Illustration of the steps for in vivo hypernociception assay.

Carrageenan was administered locally (hind paw, intraplantar—i.pl.) at a volume of 100 μL per paw; E-latex or E-NPs were delivered orally (p.o.). The E-latex or E-NP pre-treatment effects (60 min before) were tested against carrageenan-induced mechanical sensitization. The doses of E-latex (3 and 30 mg×Kg^-1^), E-NP (3, 30 and 100 mg×Kg^-1^) and carrageenan (100 μg) were derived from previous studies [37] and pilot experiments. E-latex was diluted in ethanol; E-NPs were diluted in water and carrageenan in saline, immediately before use. The mechanical sensitizing effect of carrageenan was evaluated 3 hours after its administration (Fig 2) [3, 24].

### Statistical analysis

The results are expressed as standard error of the mean (SEM) of measurements made on 5 animals in each group and represent the intensity of mechanical hypernociception. Comparisons across three or more treatments were made using one-way ANOVA with post hoc Tukey *t*-tests (Prism 8.0, GraphPad, San Diego, USA). p < 0.05 was considered statistically significant.

## Results and discussion

### Physicochemical properties of the nanoparticles

E-NP was prepared using the solvent evaporation process because this method is most efficient with drugs which are either insoluble or poorly soluble in the aqueous medium which comprises the continuous phase. The basic characteristics of the NPs prepared for this technique are presented in Table 1.

**Table 1:** Characterization data: Mean diameter, polydispersity index (PdI), zeta potential (ZP) and efficiency encapsulation (EE).

Nanoparticles	Diameter (nm)	PdI	ZP (mV)	EE(%)
Blank-NP	764 ± 39	0.470	-1.8 ± 0.09	-
E-NP (1.0 mg)	683± 22	0.132	-13.0 ± 1.00	78
E-NP (5.0 mg)	497 ± 6	0.145	-22.0 ± 6.00	75

NPs applications are predominantly governed by their properties whereby particle size and size distribution are crucial as the size can easily influence the drug loading, release, toxicity, *in vivo* distribution, particle stability, etc. In our study, the NPs displayed a mean diameter of between 497 and 764 nm. When administered intravenously, the NPs may have major limitations due to clearance by the reticuloendothelial system (RES). When the particle size exceeds 100 nm, the pharmacokinetic and biodistribution properties greatly change and they are detected in blood and organs like the spleen, lungs, liver, and kidney [33].

Typically, optimal release profiles are achieved by using microspheres with diameters in the range 10–200 μm. For particle diameters < 10 μm, there is a risk that microspheres will be phagocytosed by immune cells. On the other hand, microspheres >200 μm may cause an immune response and inflammation [38].

In addition to intravenous administration, there are other routes of administration that include oral administration, inhalation, intravenous injection, subcutaneous injection, intramuscular injection or in situ humoral injection, to deliver drug-loaded particles to target lesions, thus improving treatment and prognosis [39]. Thus, in the specific case of this work, the particles produced have adequate dimensions for oral administration.

The polydispersity index (PdI), which is a ratio that gives information about the homogeneity of the particle distribution in each system, reflects the quality dispersion within the range of 0.0–1.0. PdI values ≤ 0.1 indicate the highest quality of dispersion. Most researchers recognize PdI values ≤0.3 as optimum values; however, values ≤0.5 are also acceptable [40]. According to the literature, the NPs produced have a suitable PdI with most of the population monodisperse profile.

Zeta potential (ZP) is a physicochemical parameter that expresses the stability of a nanomaterial. Extremely positive or negative ZP values cause large repulsive forces, whereas repulsion between particles with similar electric charge prevents aggregation, and accordingly ensures easy redispersion [29, 41]. Different materials will have a different stability threshold; in the case of a polymer lattice, a minimum ZP of ± 20.0 mV is desirable. The results presented in Table 1 indicate that the increase in latex concentration causes a reduction in the diameter of the NP and an increase in the ZP. Since more quantity of functional groups from latex film being adsorbed to the NP surfaces, thus lowering the electrophoretic mobility, and increasing the stability compared to blank NPs. As a result, the NPs produced with 5.0 mg of E-latex reached the minimum ZP indicated for polymeric particles.

E-latex was entrapment with meaningful statistical significance as E-latex increased from 75 to 78%. Similar results were observed by Khaira et al. [42] in a study evaluating the use of nanoparticles for the delivery of the gemcitabine hydrochloride drug, where it was observed that the increase in the polymer:drug ratio resulted in an increase in the %EE in low proportions. After that, increasing the concentration of gemcitabine, the %EE decreased; this effect was attributed by the authors to the polymer’s ability to separate. Song et al. [43] sought to enhance the incorporation of vincristine sulfate (VCR) and quercetin (QC) into PLGA nanoparticles; it was observed that the mean entrapment efficiencies of these two drugs decreased dramatically with the increase of W/O volume ratio. This phenomenon was attributed to the different interaction between drug-polymer–solvent. According to the authors, this occurred due to the number of drugs partitioned into the organic phase which reduced during emulsification; meanwhile, the drug loss increased during solvent evaporation when the W/O volume ratio increased. Moreover, when the W/O volume ratio increased, the amount of QC dissolved in the aqueous phase, resulting in less QC retention in the internal phase to interact with PLGA molecules and then lower the entrapment efficiency of QC [44].

### FTIR analysis

**Fig 3:**
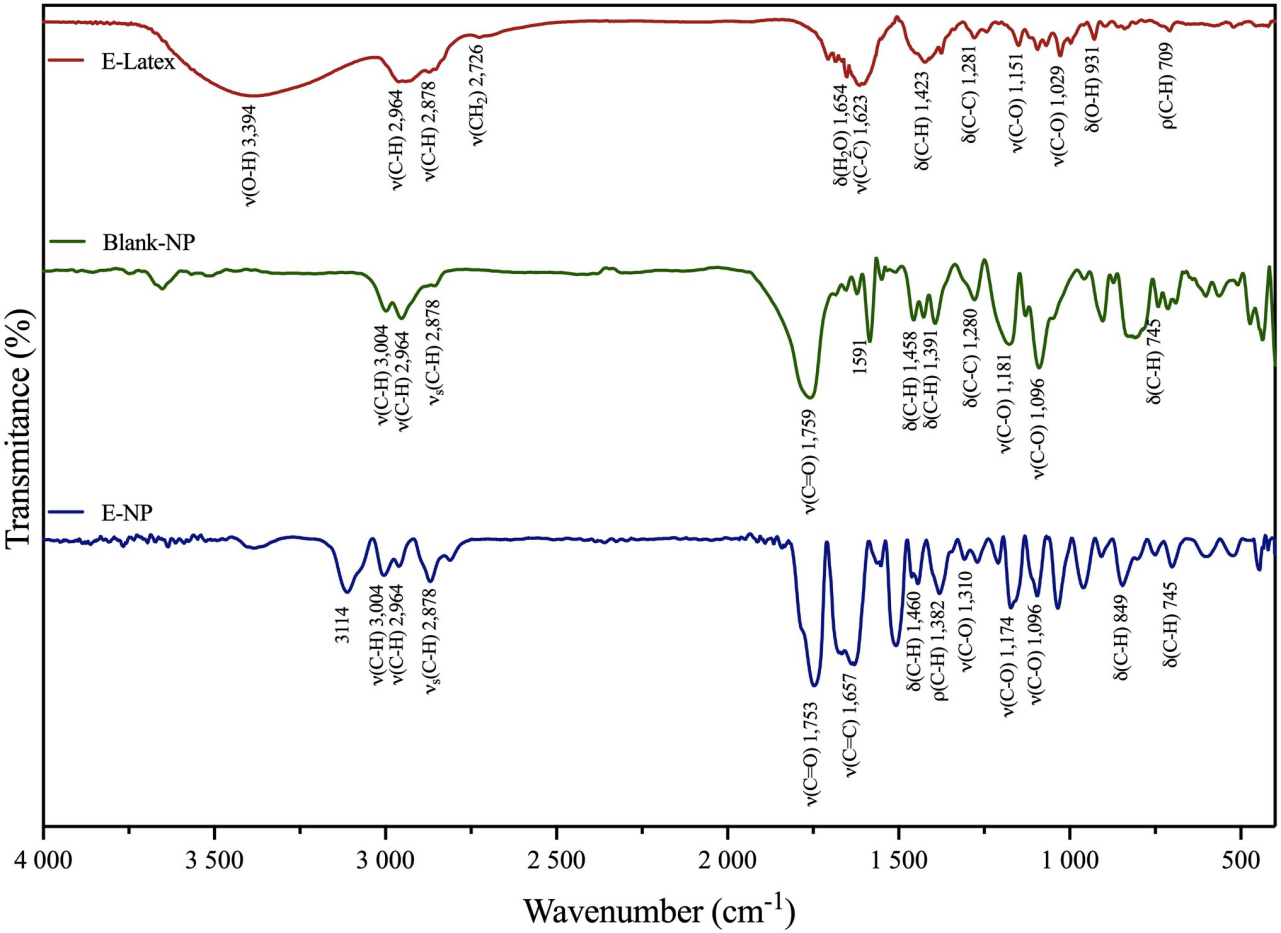
FTIR spectra of E-Latex (red line), Blank-NP (green line) and E-NPs (blue line).

FTIR is an important spectroscopic technique for chemical analysis of surface-modified NPs, revealing possible surface interactions. Our knowledge of Euphol presented in latex indicates that it belongs to the class of organic compounds known as triterpenoids, containing six isoprene units [45] and that PLGA is characterized by free carboxyl terminal groups; a covalent conjugation of Euphol with PLGA was carried out through the formation of an ester bond, which was confirmed by the observation of a strong band at 1753 cm^-1^, absence of hydroxyl (υOH) stretching vibrations of Euphol latex, which distinguishes it from the PLGA molecule. The FTIR spectra generated by E-latex, PLGA and E-NPs are represented in Fig 3.

Fig 3 display the FTIR spectra of blank PLGA nanoparticles (Blank-NP), Euphol latex (E-latex) and PLGA-Euphol conjugate (E-NP). The peaks at 3394 and 1753 cm^−1^ represent the presence of–OH and–COOH groups of PLGA. The peaks at 3121, 3004, 2964, 2878 and 2816 cm^−1^ correspond to–C-H stretching vibrations. The peak at 1753 cm^−1^ indicates the existence of C = O stretch (Ester), the two peaks at 1636 and 1657 cm^-1^ are from the Euphol latex which can be attributed to stretching -C = C- and C-C, the signals are slightly shifted to 1623 and 1654 cm^-1^, after the encapsulation process. The peaks between 1500 and 1250 cm^-1^ indicate symmetric angular deformation of the CH_3_ and CH_2_ functional groups. Between 1500 and 1250 cm^-1^ CH_3_ and CH_2_ symmetric angular deformation predominates, between 1350 and 1150 cm^-1^ asymmetric angular deformations are present from the CH_2_ and CH groups.

### Differential Scanning Calorimetry (DSC)

**Fig 4:**
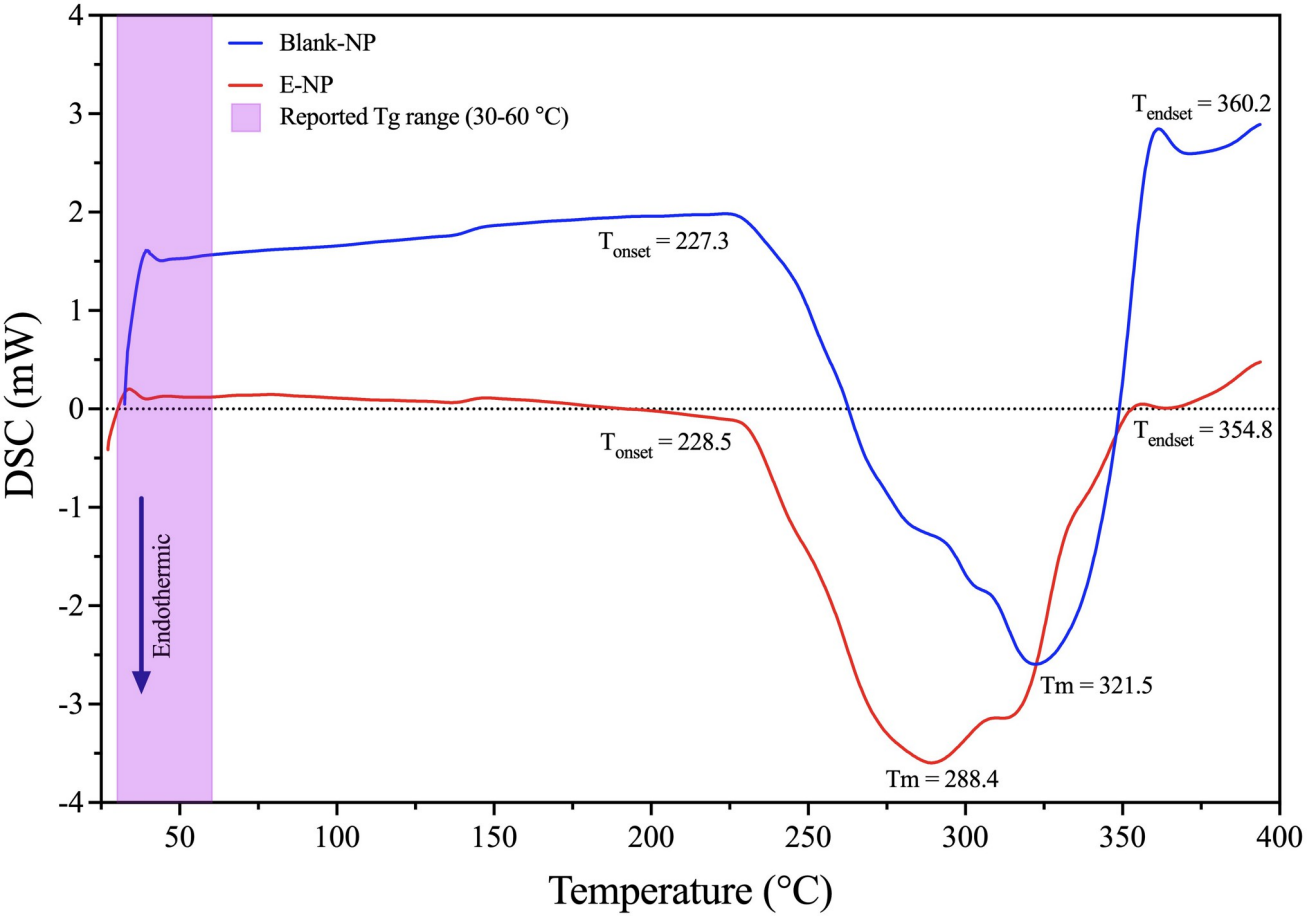
DSC analysis of PLGA NPs: Blank NP (blue line) and E-NPs (red line).

DSC is a powerful tool for the analysis of polymer-drug interactions and has previously been used to show that the drug and polymer are molecularly dispersed [41]. PLGA polymer exhibits glass transition temperatures (Tg) from 30–60°C according to literature, which is above the physiological temperature, and these provides it a sufficient strength to be used in drug delivery device [44–46]. In addition, the Tg data from PLGA 50:50 is the lowest (35.7°C) value when compared with other PLGA polymers ratios [46]. When considering properties of substances at a nanoscale level, it is expected that the properties will be different from those of the bulk material, often because of the greater surface-to-volume ratio the nano substances [46]. The Tg data range from DSC curves analyzed in both PLGA nanoparticles (Blank and E-latex loaded) were in accordance with reported literature range as displayed in Fig 4 [46]. Also, the thermal decomposition of both PLGA nanoparticles start at 227°C and ends at 360°C, which is lower than that of the PLGA polymer alone (320°C) [46]. This behavior can be attributed to the fact that the NPs are more exposed to thermal degradation because their sub-micrometric size makes the superficial area larger. As the nanoparticles have a larger surface region, they degrade more easily in comparison to the polymer [44]. However, the melting behavior of PLGA NP changes when it is in Blank, or E-latex loaded (Tm = 321.7°C and Tm = 288.4°C) respectively. Thus, the E-latex incorporated into the NPs, can be in an amorphous or disordered-crystalline phase of molecular dispersion in the polymer matrix. Similar results with other hydrophobic drugs encapsulated in the PLGA matrix were obtained by other authors [26, 46–50]. TGA was used to determine the weight loss of all samples as the temperature increases gradually to 400 °C in the presence of Nitrogen gas and E-NP lose about 89.5% of their weight at temperatures below 300 °C owing to the loss of water at the nanoparticle’s surface and water bound inside the E-NPs.

### *In vitro* drug release profile and kinetic analysis

**Fig 5:**
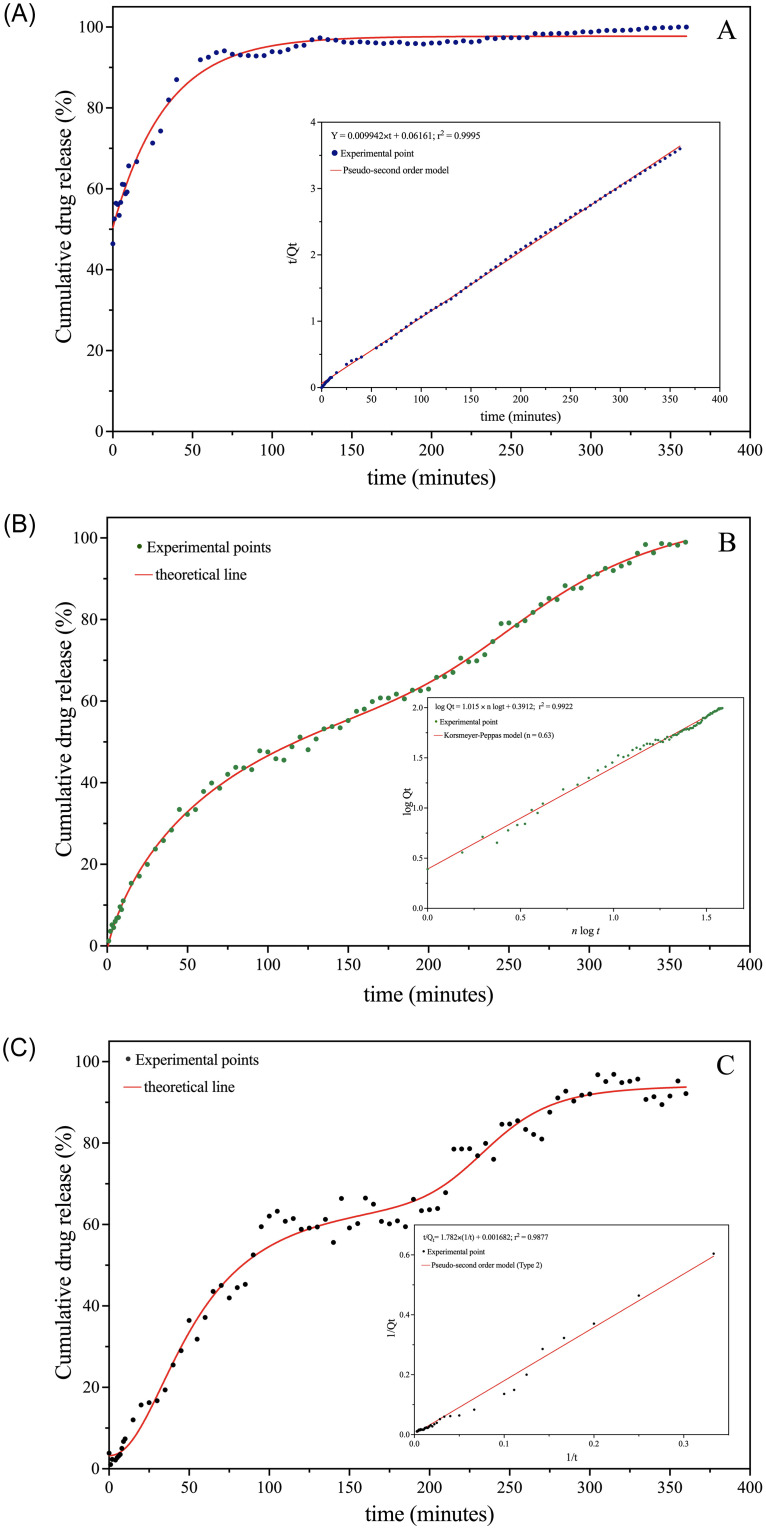
Experimental release profile of E-NP: A) pH 1.0 (dark blue dot), (B) pH 7.4 (green dot) and C) pH 8.5 (black dot), red curve indicate the best fitting curve. Insert: kinetic model graph.

The calculation of the theoretical release profile is important to assess the formulation against release rates and to confirm that it releases the test compound in a predetermined manner according to the theoretical release pattern [31, 32]. The *in vitro* drug release study was carried out for a period of 6.0 h at pH 1.0, 7.4 and 8.5, until the concentration of the E-latex released in the solution reached saturation (Fig 5A–5C). These pH values were chosen due to oral administration of drug-loaded NPs exposes them to different pH environments in the gastrointestinal tract, ranging from highly acidic (pH 1.6–3.2 in the stomach) to alkaline (pH 7.5–8.0 in the small intestine) [44]. To investigate the mode by which the E-latex molecules gradually diffuse from the matrix, the release data were analyzed with the following drug release kinetic approaches such as zero-, first-, second- and third-order, the kinetic models of Higuchi, Korsmeyer-Peppas, Weibull, Hixson-Crowell, Michaelis-Menten, and the Hill equation [31, 32]. These mathematical models consider various physical processes such as dissolution, diffusion, partitioning, osmosis, swelling, and erosion [33]. In this work, the accurate adjust plots of kinetic models are shown in Fig 5A–5C(Insert).

In the studies performed at acidic medium at pH 1.0, it was observed the release profile of Euphol latex from PLGA nanoparticles follows an exponential behavior (blue dots), as shown in Fig 5A. The theoretical curve that best fits the experimental points is shown in Eq 15 with a correlation coefficient of 0.9903.


$$Q_{t}=97.79-\left(\left(51.49\right)\times e^{\left(-0.02835\times t\right)}\right)$$ (15)

At highly acidic conditions, it was observed that approximately 91% of the compound was released after 45 minutes; after this time E-latex reach out the polymer matrix slowly and sustainably, plateauing after 130 minutes.

This is attributed to the process of erosion of the matrix and subsequent release of the drug. PLGA degradation occurs through a process of autocatalytic hydrolysis of the ester bonds in the polymer chains due to protonation of its carboxylic acid group (pKa = 3.85) [51, 52]. Each hydrolyzed ester linkage forms one hydroxyl and one carboxylic acid group, which leads to the production of acidic oligomers that catalyze the further degradation of the parent polymer. These acidic oligomers are finally hydrolyzed to lactic and glycolic acids [53, 54]. Similar results were obtained by Jain [55] for biomedical applications of biodegradable polyesters and Wan [53] for resveratrol-loaded PLGA nanoparticles.

As may be observed through Fig 5A (Insert) at pH = 1.0 the Euphol latex release follows the Pseudo-second order kinetic model, which is mathematically fitted with Eq 16, with a correlation coefficient equal to 0.9995.


$$\frac{t}{Q_{t}}=0.009942\times t+0.06161$$ (16)

In the studies performed at physiological pH 7.4 (green dots) profile it was observed that the best mathematical fit (red line) to the release profile was the two-phase model which can be seen in Fig 5B and evidenced by Eq 17, which showed a correlation coefficient of 0.9994.


$$Y=-0.0180+\left(\frac{109.0\times t^{0.77}}{t^{0.77}+43.0}\right)+\left(\frac{27.4\times t^{8.22}}{t^{8.22}+7.2}\right)$$ (17)

At pH 7.4, a quick release is observed in the first 80 minutes, which is attributed to release of E-latex adhering release to the surface of the NPs. The initial burst is followed by a plateau in a short time of between 85 and 105 minutes, then the release of E-latex follows zero-order kinetics up to 335 minutes when 98.4% of the compound has been released; in the last 25 minutes of monitoring, a plateau is observed. The *in vitro* release study revealed that at alkaline conditions, 62% of the E-latex is released in the first 105 minutes. In the time interval between 105 and 175 minutes, no significant variation, with approximately 3.0% release, is observed, which can be characterized as a plateau; in the sequence, a sustained release with a few experimental oscillations was observed because of its slow diffusion. After 1.0 h, the amount of E-latex released from the NPs was significantly higher at pH 1.0 than at pH 7.4 and pH 8.5. The amount of E-latex released from the matrix at 1.0 h was estimated to be 92.5% at pH 1.0, 37.7% at pH 7.4, and 39.6% at pH 8.5, indicating that E-NPs followed pH-dependent release kinetics [56, 57]. In the insert of Fig 5B, the kinetic modeling data at pH 7.4 the Korsmeyer-Peppas model (Eq 8) showed better linear fit regression coefficient value of r^2^ = 0.9922 and a value of *n* = 0.6294 ± 0.0069 this result and the value of *n* were determined by plotting log (fractional release of drug) versus log *t* [33].


$$logQ_{t}=0.3912+1.015\times n\;logt$$ (18)

This model is generally used to analyze the release of pharmaceutical polymeric dosage forms when the release mechanism is not well-known or when more than one type of release phenomenon could be involved [58]. It is important to note that depending on the value of *n* that better adjusts to the release profile of an active agent in a matrix system, it is possible to establish a classification, according to the type of observed behavior. Peppas et al. used this *n* value to characterize different release mechanisms: Fickian diffusion (*n* = 0.43), Anomalous transport (0.43 < *n* < 0.85), Case I transport (n = 0.85), Super Case II transport (*n* > 0.85) [33, 58]. In the case of pH 1.0 and pH 7.4, the *n* values were above 0.43 (spherical shape) namely, 0.6294 ± 0.0069, suggesting a coupling of diffusion and erosion mechanisms, anomalous diffusion, and possibly indicating that Euphol latex release from E-NPs was controlled by more than one process. The results obtained are consistent with those described in the literature [26, 27, 32, 59], where it is observed that during the early phases, the entrapped therapeutic agent is released primarily through diffusion within the polymer matrix, whereas during the later phases, the compound is released via both diffusion and degradation of the polymer matrix.

At alkaline pH 8.5 the release profile of Euphol latex also showed a biphasic behavior (black dots) as shown in Fig 5C. Eq 19 describes this behavior with a correlation coefficient equal to 0.9961.


$$Y=0.3614+\left(\frac{65.28\times t^{2.484}}{t^{2.484}+19254.18}\right)+\left(\frac{34.86\times t^{9.623}}{t^{9.623}+1.33E23}\right)$$ (19)

Under neutral or alkaline conditions, PLGA can undergo several terminal modifications, such as favoring the conversion of the free carboxylic acid terminal group to an ester terminus, which considerably affects the final physicochemical characteristics. It is reported in the literature that the mucoadhesive properties of PLGA increase the residence time of the drug-laden delivery system, improving its oral absorption [44]. A delay in degradation time of 4 to 6 weeks has been found *in vivo* for an ester end-capped PLGA in comparison with a more hydrophilic acid-terminated PLGA of a similar molecular weight and co-polymer composition [55]. As shown in Fig 5C-insert, in the experiments performed at pH = 8.5 it was observed that the best mathematical fit follows the Pseudo-second order Type 2 model (Eq 20), which showed a correlation coefficient r^2^ = 0.9877.


$$\frac{t}{Q_{t}}=1.782\left(\frac{1}{t}\right)+0.00168$$ (20)

### *In vivo* hypernociceptive assay of E-latex and E-NPs

As described before, isolated Euphol latex showed an antinociceptive effect on mice after oral administration [17, 60, 61]. The encapsulation of E-latex in PLGA NPs aims to preserve the antinociceptive effect as compared to its free E-latex form and preserve the compounds from the degradation process. The pre-treatment with E-NPs (30 or 100 mg/Kg) p.o. (%O 11.150 ± 1.622, n = 5; %O 11.068 ± 2.701, n = 5, respectively) or E-latex (30 mg/Kg) p.o. (%O -0.178 ± 1.513, n = 5) significantly inhibited the carrageenan-induced mechanical hypernociception. It was the first time that antinociceptive effects for E-NP were described and it was in accordance with data from the E-latex control as well as similar data from the literature [62].

**Fig 6:**
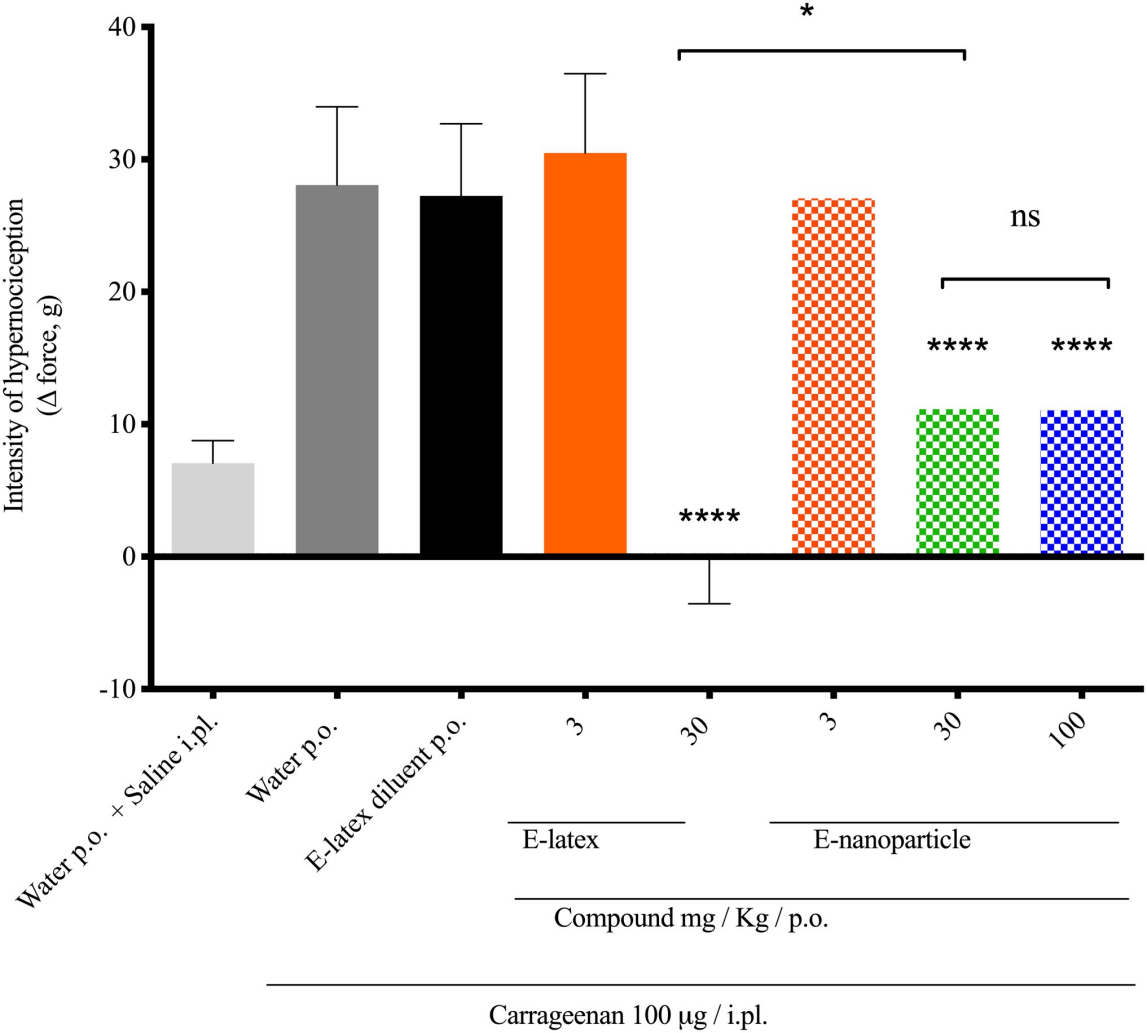
Carrageenan-induced hypernociception in rats is inhibited by the free or encapsulated compound.

The comparison between both E-latex and E-NP intensity of effects were shown to be significantly different. Once administered, drug release is an important parameter, to know how the system will maintain the concentration of the compound in the target tissues at a desired value as long as possible, exerting a control on the drug release rate and duration. The effect of pH on the release profile was observed, mimicking the release in gastrointestinal fluid (pH 1.0), in the small intestine and blood (pH 7.4) and in the colon (pH 8.5). The results indicate that the E-NPs followed pH-dependent release kinetics with a quick exit of the compound under acidic conditions. Pharmacokinetic as well pharmacodynamic properties conferred by nanoencapsulation of E-latex can be explored in future studies. The data demonstrate the antinociceptive effect for E-NP, suggesting that E-latex nanoencapsulation preserved its desired properties Fig 6.

The Fig 6 shows the hypernociception induced by a single i.pl. injection of carrageenan (100 μg/paw) and the effect of pre-treatment with the free (3 and 30 mg/Kg/p.o.) or encapsulated compound (3, 30 and 100 mg/Kg/p.o.) or water. The data are expressed as mean SEM; n = 5. ****p < 0.0001 compared to water plus the carrageenan group; *p < 0.05 compared to E-latex plus the carrageenan group; one-way ANOVA with Tukey’s comparison.

## Conclusion

The results of this study revealed that E-NPs were prepared successfully using the solvent evaporation method with an encapsulation efficiency of ≥75%. FTIR demonstrated the polymer and Euphol latex interaction. The physicochemical characteristics of this release system such as hydrodynamic diameter, zeta potential and physical stability up to 37°C, as well as the biocompatibility and biodegradability of the polymer indicate that E-NPs are suitable for oral delivery due to versatile biodegradation in distinct pH. Animal assays with E-NPs or E-latex significantly inhibited the mechanical hypernociceptive induced by carrageenan, suggesting that the nanoencapsulation process of Euphol latex is impaired during the entrapment and administration process. Further studies are needed to explore the use of nanotechnology for the possible antinociceptive effect induced by E-latex.
